# Cancer-Associated Fibroblast-Derived IL-6 Determines Unfavorable Prognosis in Cholangiocarcinoma by Affecting Autophagy-Associated Chemoresponse

**DOI:** 10.3390/cancers13092134

**Published:** 2021-04-28

**Authors:** Suyanee Thongchot, Chiara Vidoni, Alessandra Ferraresi, Watcharin Loilome, Narong Khuntikeo, Sakkarn Sangkhamanon, Attapol Titapun, Ciro Isidoro, Nisana Namwat

**Affiliations:** 1 Department of Biochemistry, Faculty of Medicine, Khon Kaen University, 123 Mitraparp Highway, Khon Kaen 40002, Thailand; suyanee.tho@mahidol.ac.th (S.T.); watclo@kku.ac.th (W.L.); 2 Laboratory of Molecular Pathology, Department of Health Sciences, Università del Piemonte Orientale “A. Avogadro”, Via Solaroli 17, 28100 Novara, Italy; chiara.vidoni@med.uniupo.it (C.V.); alessandra.ferraresi@med.uniupo.it (A.F.); 3 Siriraj Center of Research Excellence for Cancer Immunotherapy (SiCORE-CIT), Research Department, Faculty of Medicine Siriraj Hospital, Mahidol University, Bangkok 10700, Thailand; 4 Cholangiocarcinoma Research Institute, Khon Kaen University, 123 Mitraparp Highway, Khon Kaen 40002, Thailand; knaron@kku.ac.th (N.K.); sakkarn@kku.ac.th (S.S.); attati@kku.ac.th (A.T.); 5 Department of Surgery, Faculty of Medicine, Khon Kaen University, 123 Mitraparp Highway, Khon Kaen 40002, Thailand; 6 Department of Pathology, Faculty of Medicine, Khon Kaen University, 123 Mitraparp Highway, Khon Kaen 40002, Thailand

**Keywords:** cholangiocarcinoma, cancer-associated fibroblasts, interleukin-6, cytokines, autophagy, desmoplastic stroma, cancer therapy

## Abstract

**Simple Summary:**

We aimed to validate with clinical and molecular data the hypothesis that CAF infiltration and release of IL-6 predict poor prognosis in CCA patients following dysregulation of autophagy in cancer cells. Stromal IL-6 and cancer-cell-associated autophagy proteins were assayed by Tissue MicroArray immunohistochemistry and their expression correlated with overall survival (OS) in a cohort of 70 CCA patients. We found that patients bearing a CCA with low stromal IL-6 and active autophagy flux in the cancer cells have the best prognosis and this correlates with a more effective response to post-operative chemotherapy. A similar trend was observed in CCA patients from the TCGA database. In vitro experiments with primary CAFs isolated from human CCA showed that IL-6 impairs the autophagy-associated apoptotic response to 5-FU in human CCA cells. Stromal IL-6 inhibition of autophagy in cancer cells was confirmed in an animal model of CCA. Our data support a therapeutic strategy that includes drugs limiting the stromal inflammation and enhancing autophagy to improve the survival of CCA patients.

**Abstract:**

Background: Interleukin-6 (IL-6) released by cancer-associated fibroblasts (CAFs) has been shown to associate with the malignant behavior of cholangiocarcinoma (CCA). Here, we aimed to validate with clinical and molecular data the hypothesis that CAF infiltration and release of IL-6 predict poor prognosis in CCA patients following dysregulation of autophagy in cancer cells. Methods: Stromal IL-6 and cancer-cell-associated autophagy proteins LC3 and p62 were assayed by Tissue MicroArray immunohistochemistry and their expression correlated with overall survival (OS) in a cohort of 70 CCA patients. The 5-FU cytotoxicity and autophagy were determined in CCA cells cultured with CAF-conditioned medium. Results: We show that patients bearing a CCA with low production of stromal IL-6 and active autophagy flux in the cancer cells have the best prognosis and this correlates with a more effective response to post-operative chemotherapy. A similar trend was observed in CCA patients from the TCGA database. In vitro genetic manipulation of IL-6 production by primary CAFs isolated from human CCA showed that IL-6 impairs the autophagy-associated apoptotic response to 5-FU in human CCA cells. Stromal IL-6 inhibition of autophagy in cancer cells was confirmed in an animal model of CCA. Conclusion: Our data support a therapeutic strategy that includes autophagy-enhancing drugs along with adjuvants limiting the stromal inflammation (i.e., the secretion of IL-6) to improve the survival of CCA patients.

## 1. Introduction

Cholangiocarcinoma (CCA), the cancer of bile duct epithelia, is the second most common primary malignancy of the hepato-biliary system, and its incidence rate has significantly increased over the last few decades worldwide [1,2]. CCA remains a major concern, especially in Southeast Asia, where the pathogenesis is essentially associated with chronic liver fluke infection [3,4]. Otherwise, it is a deadly disease with a 9% mortality ratio within three months [5], and surgical resection followed by radio- and/or chemotherapies offers survival rates of approximately 20–40% and 5–10% at 5 years and 10 years, respectively [6,7]. Surgery and chemotherapy (usually with gemcitabine, cisplatin, or 5-FU) elicit however very modest success [8]. Unfortunately, a late diagnosis often precludes the possibility of surgical intervention. Additionally, even in this case recurrence and progression with emergence of polychemoresistance are very common, which explains the very poor prognosis of CCA [9,10,11].

In the last few decades, gene profiling [12,13,14,15] and clinical histopathology [16,17,18,19,20] studies have greatly added to our knowledge of the genes and molecular pathways that are involved in the pathogenesis and progression, and define the prognosis, of CCA. The top genes that were found to be abnormally expressed in CCA, with a frequency ranging from 10% to >50 depending on the topographical location (intra or extrahepatic), the population studied, and the method include *TP53*, *KRAS*, *CDKN2/p16^INK4^*, *FGFR2* gene fusions, *ERBB2*, *IDH1*, and *ARID1A* [12,15,21]. The main molecular pathways that were found to be altered in CCA include the chromatin rearrangement, epigenetic regulation, proliferation signaling, apoptosis, and DNA repair [21,22,23]. In addition, there is some evidence for involving autophagy in cholangiocarcinogenesis [24,25,26]. Autophagy, the lysosomal-driven macromolecular degradation pathway that plays a major role in tissue homeostasis, is known to be dysregulated in cancer cells subject to changes in the tumor microenvironment in terms of nutrients, oxygen, growth factors, cytokines, and other signaling molecules [27,28,29]. Recently, we have shown that IL-6 secreted by primary cancer-associated fibroblasts (CAFs) isolated from human CCA is capable of inhibiting autophagy in CCA cells and by doing so it stimulates their proliferation and invasive potential [30]. It remains to be determined whether such an effect is indeed happening in CCA-bearing patients and, if so, whether it has any prognostic value and significance for therapeutic intervention.

In this work, we address these questions, adding clinical evidence and in vitro mechanistic explanations in support of the view that CAF infiltration indeed causes CCA progression through impairing the autophagy flux in cancer cells, thus resulting in reduced chemoresponsiveness. That stromal CAFs and IL-6 inhibit autophagy in cancer cells was further confirmed in an experimental model of CCA resembling human liver fluke-induced cholangiocarcinogenesis. Our experimental data, together with bioinformatic analysis of TCGA data, demonstrate the relevance of IL-6 and of autophagy proteins as prognostic markers in CCA patients. We also show that IL-6 is involved in the autophagy-mediated cytotoxic response to the chemotherapeutic drug 5-FU. The present data have therapeutic implications supporting the inclusion of autophagy-enhancing drugs along with adjuvant therapies capable of dampening stromal inflammation for better management of CCA.

## 2. Results

### 2.1. Clinicopathological Data of CCA Patients

Seventy cases of CCA tissue microarray (TMA) liver sections, from male (62%) and female (38%) patients aged between 32 and 82 years old (median = 60 years old), with complete clinicopathological data were included in the study. Tumors with low and high extension of fibrotic areas were selected. A pathological examination confirmed that all cases were intra-hepatic adenocarcinomas from bile ducts. All 70 cases were subjected to surgical resection. Chemotherapy was administered to 6 patients before and to 24 patients after surgery, while 40 patients were not subjected to any chemotherapy. During a median of 13.6 (range, 0.0–41.1) months of follow-up, 34 of the 70 patients (48.57%) died. Follow-up information was available for the 36 patients surviving up to 41.1 months. The clinicopathological characteristics of CCA patients, including age, sex, tumor staging, tumor size, tumor node metastasis (TMN), histological grading, and chemotherapy with respect to the level of LC3 and p62 expression in cancer cells and of IL-6 in CAFs are presented in Table 1.

### 2.2. IL-6 Expression in Fibrotic Stroma Associates with Poor Prognosis in CCA Patients

**Figure 1:**
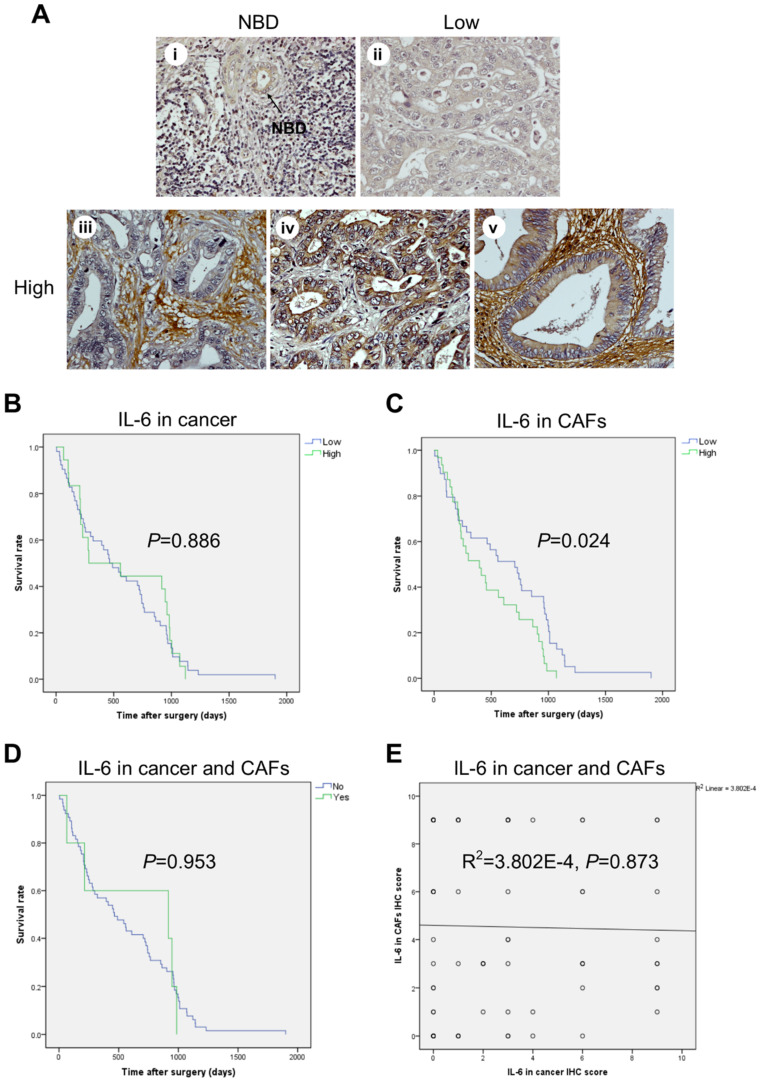
High expression of IL-6 in fibrotic stromal cells significantly correlates with shorter survival in CCA patients. (**A**) Representative images of IL-6 immunohistochemistry (IHC) in human CCA tissues showing faint staining (negative) in normal bile ducts (NBDs) (**i**) and in some cancer areas (**ii**), while showing strongly intense (positive) staining in fibrotic areas rich in CAFs (**iii**), in a few cancer areas (**iv**), and in both cancer epithelial cells and fibrotic areas (**v**). (**B**–**D**) Kaplan–Meier survival analysis for IL-6 IHC staining in cancer areas (**B**), or in CAF areas (**C**), or in cancer and CAF areas (**D**). (**E**) The relationship between IL-6 protein expression in cancer and CAFs is illustrated as scattered plots with linear regression lines. (Magnification 40×).

Immunohistochemical (IHC) staining of IL-6 and of the autophagy markers LC3 and p62 was performed and the staining intensity scored in epithelial and in stromal (namely, CAF) cells. Faint to negative staining of all the three proteins tested was found in normal bile duct epithelia (Figure 1A). In contrast, CAFs and cancer cells showed positivity, yet to a different extent. On the whole, the percentage of positive cells in human CCA tissues was 50% for IL-6, 24% for LC3, and 44% for p62. The IHC for IL-6 expression in the cytoplasm was scored separately for epithelial cancer cells and in fibrotic (CAF-enriched) areas (Figure 1). The staining ranged from absent (score 0) to strong (score 3) in CCA tissues. Twenty four of 70 cases (34%) were positive in only fibrotic areas (Figure 1A(iii)), 7 cases (10%) were positive in cancer epithelial cells (Figure 1A(iv)), and 39 cases (56%) were positive in both cancer epithelial cells and fibrotic areas (Figure 1A(v)). No significant differences between staining patterns and clinicopathological features, such as age, sex, presence of lymph node metastasis or distant metastasis, grading, and tumor location and size, were observed (Table 1). Fisher’s exact test indicated a significant inverse correlation of IL-6 positive staining in cancer cells and fibrotic areas and the drug regimen status (Table 1, *p* < 0.025). Further, and most importantly, survival analysis demonstrated that high IL-6 in CAF-containing fibrotic areas was significantly associated with a shorter overall survival time in univariate (Figure 1C; I = 0.024) and multivariate analyses (Table 2; HR = 2.004; CI = 1.138–3.527; *p* = 0.016).

### 2.3. High Expression of LC3 and Low Expression of p62 in CCA Cells Correlate with Better Prognosis

**Figure 2:**
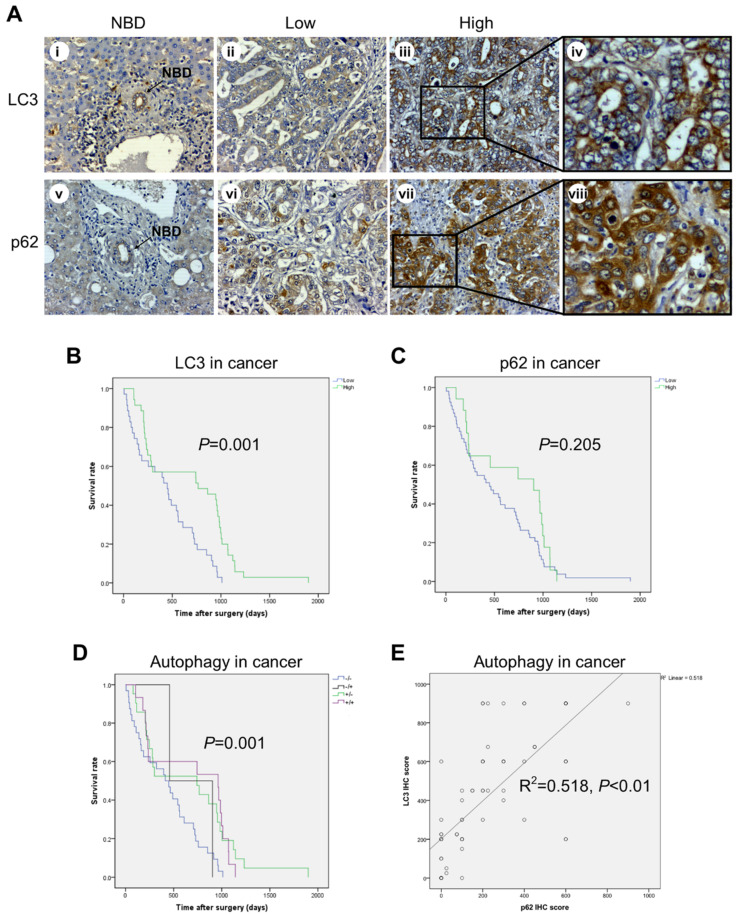
Immunohistochemical expression of LC3 and p62 in cancer cells and its correlation with overall survival of CCA patients. (**A**) Representative cases showing LC3 and p62 immunostaining in normal bile ducts (NBDs) (panels i and v) and in cancer areas (panels **ii**–**iv** and **vi**–**viii**). Note that p62 staining is at all times higher than that of LC3 in the cancer area considered. Original magnification is x200. (**B**–**D**) Cumulative survival analysis shows a significantly longer survival rate for CCA patients with high LC3 and low p62 expression, indicative of ongoing autophagy. (**E**) The relationship between LC3B and cytoplasmic p62 expression exhibits a positive correlation. (Magnification 20× and 40×).

Given the potential involvement of autophagy in CCA progression [24,25,26,31,32,33], we sought to assess the IHC expression profile of the autophagy proteins LC3 (a marker of autophagosome) and p62/SQSTM1 (a marker of the autophagy cargo) in CCA TMAs (Figure 2). The high IHC score of LC3 (Figure 2A) showed a positive association with longer overall survival of CCA patients in univariate (Figure 2B; green line; *p* = 0.001) and multivariate analysis (Table 2; HR = 0.401; CI = 0.236–0.681; *p* = 0.001). Of note, no significant associations between p62 IHC staining and clinical outcome were found (Figure 2C). Consistently, the prognosis was better in patients bearing a CCA with high expression of LC3 along with low (green line) or high (violet line) p62 expression compared with patients bearing a CCA with low expression of LC3 (Figure 2D). Spearman’s correlation test was performed to examine the relationship between LC3 and p62 expressions in epithelial cancer cells (Figure 2E). The pattern of high LC3 but low p62 showed a positive correlation in CCA tissues (Figure 2E; rho = 0.518; *p* = 0.000), supporting the view that high LC3 was reflecting effective autophagy degradation of the cargo. Remarkably, the combined pattern of high LC3 and low p62 showed a significant correlation with the best overall survival in univariate (Figure 2D; green line; *p* = 0.001) and multivariate (HR = 2.344; CI = 1.222–4.496; *p* = 0.01) analysis.

### 2.4. Correlation between IL-6, LC3, and p62 Expression and Clinicopathologic Features of CCA Patients

At this point, it was mandatory to check whether inflammation (marked as IL-6 production in fibrotic stroma) and autophagy (marked as LC3 up-expression and p62 down-expression in epithelial cancer cells) were correlated and how the various combinations would correlate with clinical prognosis. With regard to the IHC protein expression scores, of the 70 cases, 18 cases (25.7%) were classified as low IL-6, low LC3, and low p62 (L/L/L); 11 cases (15.7%) were classified as low IL-6, high LC3, and low p62 (L/H/L); 1 case (1.4%) was classified as low IL-6, low LC3, and high p62 (L/L/H); and 9 cases (12.9%) were classified as low IL-6, high LC3, and low p62 (L/H/H), whereas 14 cases (20.0%) were classified as high IL-6, low LC3, and low p62 (H/L/L); 9 cases (12.9%) were classified as high IL-6, high LC3, and low p62 (H/H/L); 2 cases (2.9%) were classified as high IL-6, low LC3, and high p62 (H/L/H); and, finally, 6 cases (8.6%) were classified as high IL-6, high LC3, and high p62 (H/H/H). Assuming that autophagy flux proceeds to completion when LC3 is up-expressed and p62 is down-expressed, the autophagy flux was clearly effective in 20 cases, of which 11 presented with low and 9 presented with high expression of IL-6. These numbers do not allow us to draw any convincing correlation between the level of IL-6 in the stroma and the level of autophagy in cancer cells.

**Figure 3:**
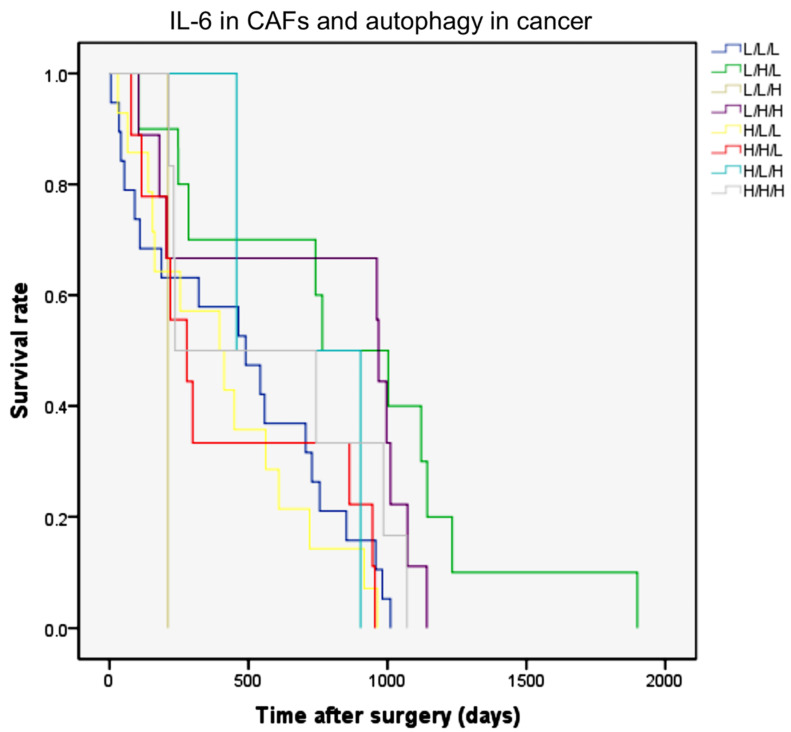
The Kaplan–Meier curves of overall survival rates according to IL-6 in fibroblasts and LC3 and p62 expression in CCA epithelial cells. The combination of IHC expression of IL-6 in stromal fibrotic areas and of autophagy markers (LC3 and p62) in cancer cells were classified into eight patterns as indicated in the text. Combined low IL-6, high LC3, and low p62 (L/H/L) expression significantly correlates with better overall survival rates (green line; *p* = 0.007, log-rank test).

Next, we calculated the overall survival for the patients classified according to the above combinations. The Kaplan–Meier curves are shown in Figure 3. It was found that the pattern of L/H/L, representing a low inflammatory stroma (low IL-6 staining) and an efficient autophagy flux in cancer cells (high LC3 and low p62), was significantly associated with the best prognostic clinical outcome (Figure 3, green line; *p* = 0.007). In the multivariate analysis, this scoring pattern was an independent and a significant variable that predicted a favorable prognosis. The hazard ratio (HR) for death based on this variable was 2.535 (95% confidence interval (CI) 1.122–5.727; *p* = 0.025). The pattern with low IL-6 in the stroma and high LC3/high p62 in cancer cells (violet line; L/H/H; HR: 1.659, 95% CI: 0.813–3.384) also showed a good prognosis when compared with the other patterns having high IL-6 and/or low LC3 expression.

### 2.5. The Role of Adjuvant Chemotherapy and of Inflammatory and Autophagy Marker Expression in Patient Survival

**Figure 4:**
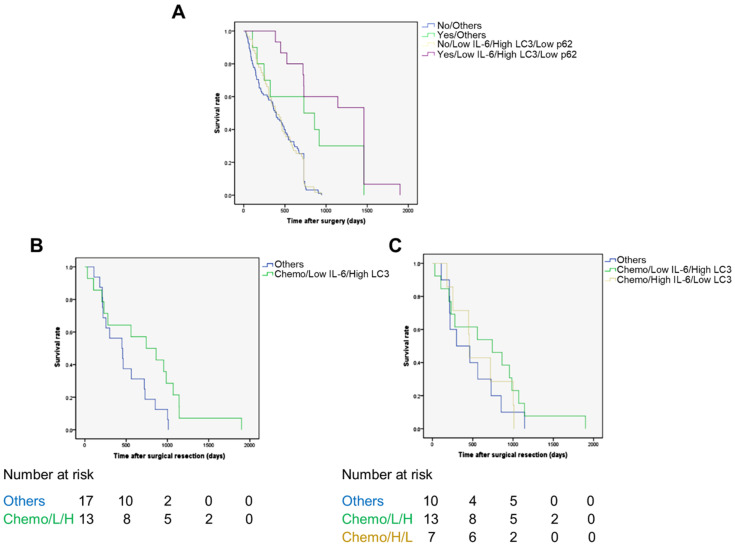
The Kaplan–Meier curves for overall survival rates according to adjuvant chemotherapy status, IL-6 in fibrotic areas, and LC3 and p62 expression in cancer. The 70 patients were categorized based on having been or not subjected to chemotherapy and the respective level of expression of Il-6, LC3, and p62 as detailed in Figure S1 (see also the text for details). (**A**) It is apparent that those patients who received adjuvant chemotherapy and bearing a CCA with a protein pattern of low IL-6, high LC3, and low p62 (L/H/L) expression (purple line) had an overall survival rate that was much better than any other combination; *p* = 0.01. (**B**,**C**) In the cohort of 30 patients subjected to chemotherapy, the group bearing a CCA with high LC3 and low stromal IL-6 (regardless of p62 expression) showed a better prognosis with respect to the group with other combinations (panel B, green line vs. blue line; *p* = 0.001, log-rank test) and to the groups bearing a tumor with high stromal IL-6 and low cancer cell LC3 (panel C, green line vs. yellow line; *p* = 0.018, log-rank test) or a tumor with any other combination (panel C, green line vs. blue line; *p* = 0.018, log-rank test).

Next, we assessed how and whether postoperative chemotherapy had affected the patient survival depending on the inflammatory and autophagy levels in the cancer. Thirty patients received chemotherapy (6 before and 24 after surgery), which included gemcitabine for 8 patients (26.7%; 12.29% of total), cisplatin for 7 patients (23.3%; 10.61% of total), and 5-FU for 15 patients (50%; 21.33% of total). In an attempt to clarify the respective role of chemotherapy versus CCA inflammation/autophagy status in the clinical outcome, we estimated the overall survival in those patients with a favorable status (based on the above analysis), i.e., with low IL-6, high LC3, and low p62, that were subjected or not to chemotherapy (see Figure S1). Additionally, to determine whether the chemotherapy per se affected the clinical outcome, we have also estimated the overall survival for the other patients. The Kaplan–Meier curves are shown in Figure 4. It is clearly apparent that patients who could not benefit from chemotherapy had the poorest outcome (Figure 4A). Strikingly, those patients bearing a CCA with low IL-6 in fibroblasts and a high LC3 and low p62 pattern in cancer cells (L/H/L) were the ones most benefiting from the adjuvant chemotherapy. This outcome was statistically significant (Figure 4A, purple line; L/H/L; *p* = 0.01). The multivariate analysis indicated that the status of drug-treated plus low IL-6 plus high LC3 plus low p62 was an independent factor associated with longer overall survival. Overall, we found a positive correlation between drug-treated and low IL-6 (in fibroblasts) plus high LC3 plus low p62 in cancer cells (Table 3; Pearson r = 0.898, *p* < 0.01). Based on the previous observations, we assumed that high expression of LC3 could impact the chemoresponsivity regardless of the level of p62 expression.

To test this hypothesis, we estimated the OS in the cohort of 30 patients subjected to chemotherapy, considering the group bearing a high-LC3-expressing CCA along with low IL-6 in CAFs (regardless of p62 expression) (n = 13) vs. the other combinations (n = 17). Again, the former group showed a better OS (Figure 4B; *p* = 0.001). Finally, we compared the OS of this group (13 patients) to the group of patients bearing a tumor with high stromal IL-6 and low cancer cell LC3 (n = 7) and the group of patients bearing a tumor with other combinations (n = 10), and again the former group showed a better survival rate (Figure 4C; *p* = 0.018).

### 2.6. Expression of Autophagy Markers and Clinical Outcome in CCA-Bearing Patients from the TCGA Database

**Figure 5:**
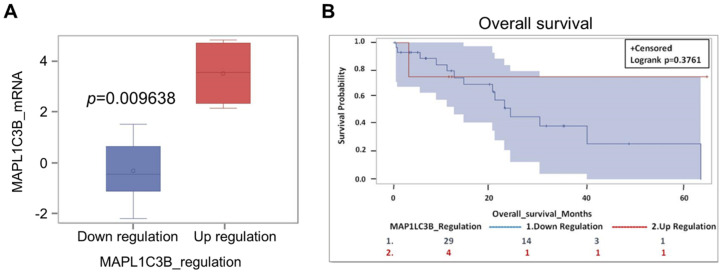
The prognostic value of autophagy marker LC3 mRNA in a cohort of CCA patients in the TCGA database. (**A**) Categorization of the CCA based on the level of MAP1LC3B (LC3) mRNA expression. (**B**) Patients bearing a CCA expressing a high level of MAP1LC3B tend to have a longer overall survival (*p* = 0.3761).

The above data refer to a cohort of patients living in a specific geographic area (a province in the North-Eastern part of Thailand) where liver fluke *Opistorchis viverrini* infection is a recognized cause of CCA. To see whether our observation could be extended to CCA cases from other countries, and likely with a different pathogenesis, we have interrogated the TCGA database. Thirty-four cases are reported in the database for which are available, with some exceptions, data on the mRNA expression and copy number variation (CNV) of IL-6 and of the autophagy genes *LC3*, *p62/SQSTM1*, and *BECN1*, along with information on overall survival (OS). *BECN1* is the first identified mammalian autophagy gene, and it is a haploinsufficient tumor suppressor coding for the BECLIN1 protein [34]. Details of this cohort of patients are provided in Table S1. The oncoprint showing the alterations in *BECN1* and *MAP-LC3B* gene expression is shown in Figure S2A. Data on mRNA expression of *LC3* were available for 33 CCA, of which 4 had high expression (two patients underwent chemotherapy) and 29 had low expression (only seven patients underwent chemotherapy). Though not statistically significant because of the small numbers of cases in the database, the trend shows that those patients bearing a CCA highly expressing LC3 have a better prognosis in terms of OS (Figure 5, panels a and b). Consistently, a better prognosis was observed in patients bearing a CCA with *MAP-LC3B* gene amplification (n = 5) compared with patients bearing a CCA with diploid CNV (n = 22) or with shallow (monoallelic) deletion (n = 6) (not shown). Data on *BECN1* mRNA expression were available for 34 patients (Figure S2B). Quite surprisingly, the 29 patients bearing a CCA expressing a low level of *BECN1* showed a better OS (not significant; *p* = 0.23) than the 5 patients bearing a CCA expressing a high level of *BECN1* (Figure S2C). It should be considered, however, that the latter patients were not subjected to chemotherapy, whereas in the group of low *BECN1* expressors nine patients were subjected to chemotherapy. Additionally, the five CCAs with high *BECN1* expressed a low level of *LC3* (Figure S2D). Interestingly, the four patients with a low *BECN1* and high *LC3* tumor (two of them underwent chemotherapy and two did not) showed a much better prognosis (not significant; *p* = 0.37) than the five patients with a high *BECN1* and low *LC3* tumor (Figure S2E). Data on *p62/SQSTM1* gene alteration and OS were available for 33 patients (Figure S3). Though not statistically significant (*p* = 0.99) because of the small numbers, we found that 29 patients bearing a CCA with a low level of *p62* (only eight underwent chemotherapy) showed a much better prognosis than the four patients bearing a CCA with a high level of *p62* (no one underwent chemotherapy) (Figure S3). Assuming that low accumulation of p62 in cancer cells is indicative of increased autophagy flux, these data are consistent with the above data on LC3. Data on *IL-6* gene alteration and OS were available for 34 patients (Figure S4), of which the three bearing a CCA with high expression showed a worse prognosis (not significant; *p* = 0.71). Unfortunately, there are no data available in the TCGA dataset for the level of IL-6 specifically expressed in the stroma.

### 2.7. IL-6 Secreted by CAFs Inhibits Autophagy and Reduces the Chemosensitivity of CCA Cells

**Figure 6:**
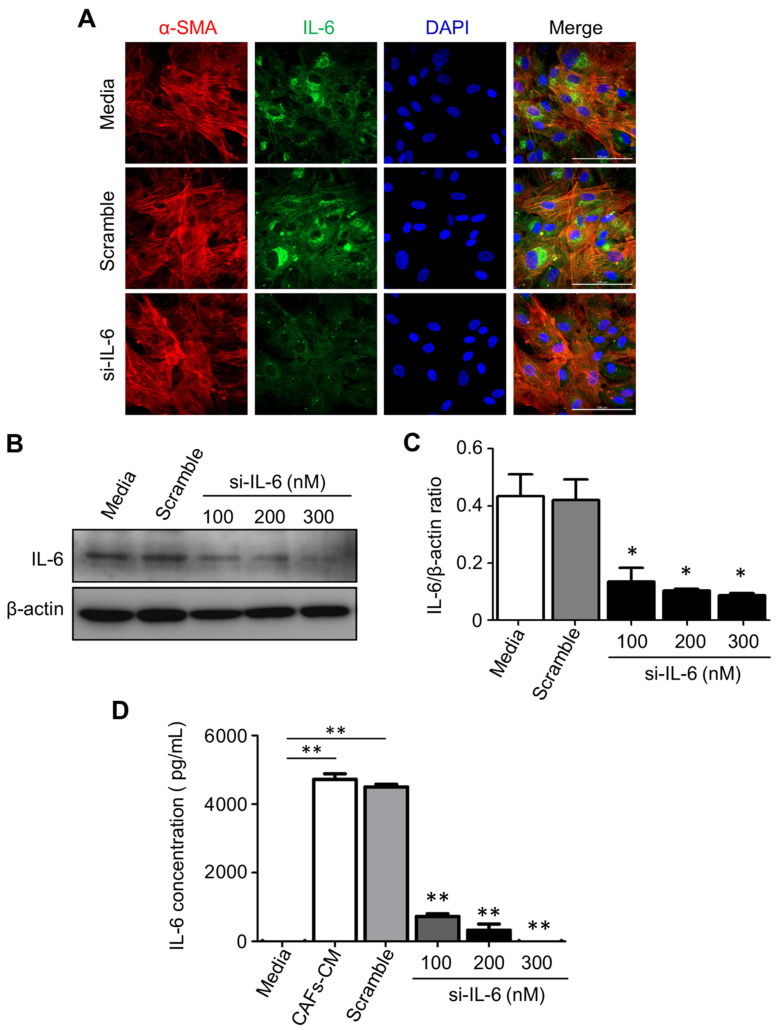
Efficacy of post-translational silencing of IL-6 in CAFs. Primary CAFs isolated from fresh CCA biopsies were transiently transfected with an IL-6-specific small interfering (si-) RNA and the efficacy of protein knockdown was tested by several methods including immunofluorescence (**A**), Western blotting (**B**), and ELISA (**C**). (**A**) The cells were stained with α-SMA (red fluorescence; a marker of CAFs), IL-6 (green fluorescence), and Hoechst (blue fluorescence; nuclei label). It is shown that si-RNA-transfected CAFs express a very low level of IL-6 (magnification 63X). (**B**) CAFs were treated with a control medium, scramble si-RNA, or 100, 200, or 300 nM of IL-6-specific si-RNA. The Western blotting shows a si-RNA dose-dependent reduction in cellular IL-6. The filter was stripped and re-probed for β-actin to verify protein loading. Densitometry is shown in (**C**). (**D**) An ELISA kit was used to analyze the levels of IL-6 in CAF CM after (or not after) treatment with scramble or IL-6-specific si-RNA. Values correspond to means ±SD of triplicates obtained in three independent experiments. * *p* < 0.05, ** *p* < 0.01, compared with the scramble.

To explain the above data, we hypothesized that IL-6 released by CAFs negatively affected the chemosensitivity of neighboring CCA cells via inhibiting the autophagy stress-response to the drug. To this end, we have specifically inhibited the production of IL-6 by transfecting the CAFs with an appropriate si-RNA. The data shown in Figure 6 prove that the si-RNA transfection effectively downregulated the expression and secretion in the medium of IL-6 by CAFs without altering their myofibroblast-like phenotype (as monitored by α-SMA expression).

**Figure 7:**
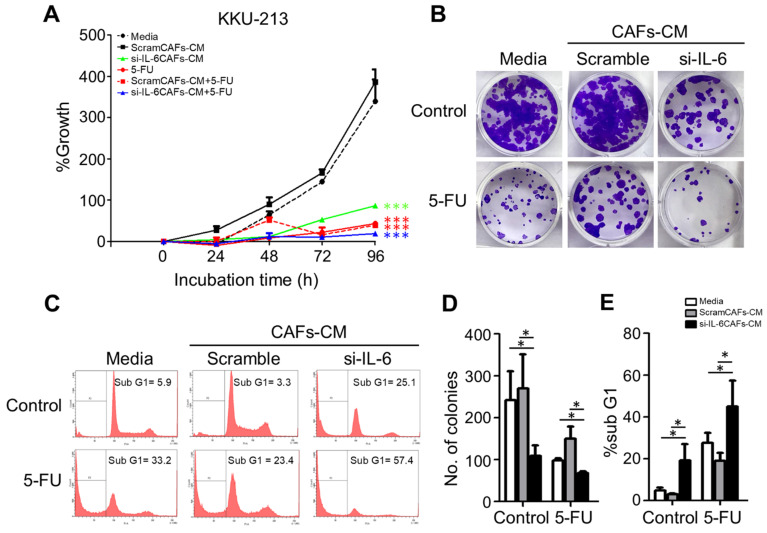
Effect of CAF medium containing IL-6 or not on the chemosensitivity of CCA cells. KKU-213 cells were incubated in a control medium or in a conditioned medium (CM) from CAFs previously transfected with scramble or IL-6-specific si-RNA and then exposed or not to the chemotherapeutic drug 5-FU as indicated in the panels. (**A**) Cell survival as determined by SRB assay after 48 h of culture. (**B**) Clonogenic assay and crystal violet staining. (**C**) Cytofluorometric analysis of the subG1 population after staining with propidium iodide (PI) of the cells treated as indicated. (**D**) The number of colonies (stained with 0.5% crystal violet) is given as mean ± SD bar of triplicate experiments. (**E**) Quantification and statistics of hypodiploid (sub-G1)-PI-positive cells representing the apoptotic population. Data represent the mean ±SD of at least three different experiments run in triplicate. * *p* < 0.05 compared to the control or medium alone.

**Figure 8:**
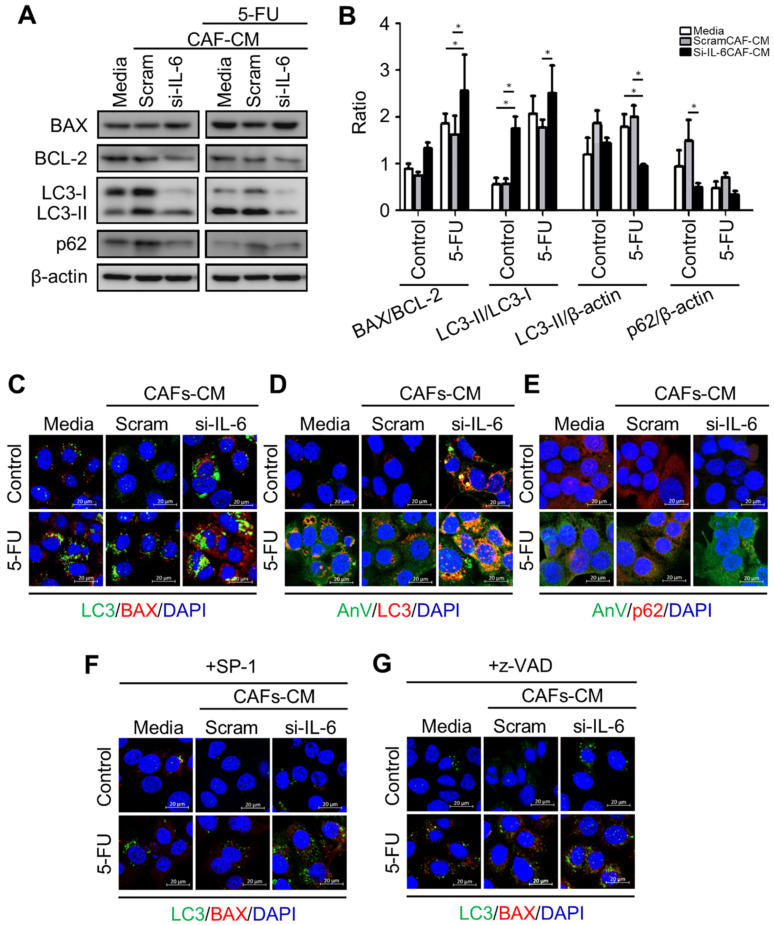
Expression of apoptosis- and autophagy-related proteins in KKU-213 cells exposed to CAF medium and 5-FU. KKU cells were incubated in a control or a conditioned medium (CM) from CAFs previously transfected with scramble or IL-6-specific si-RNA and then exposed or not for 48 h to the chemotherapeutic drug 5-FU. At the end, the expression of relevant proteins involved in apoptosis (BAX and BCL-2) and autophagy (LC3 and p62) was determined by Western blotting (panel (**A**)). (**B**) Densitometry of Western blotting data are reported as mean ± SD. (**C**–**G**) Additionally, CCA cells adherent on coverslips were incubated with media or scramble or IL-6-specific si-RNA CM and 5-FU or SP-1 or z-VAD for 24 h. The cells were fixed and immunofluorescence staining for the markers of apoptosis (BAX and Annexin V) and of autophagy (LC3 and p62) (Scale bar = 20 µM; magnification 63×). Staining with Hoechst (blue fluorescence) was done for visualize cell nucleus. Western blotting and immunofluorescence images are representative of three independent experiments. Statistical significance * *p* <0.05 compared to the control. The quantification of the immunofluorescence is reported in Figure S7.

Next, we tested whether and how the conditioned media from control or si-RNA-transfected CAFs would affect the autophagy regulation and the chemosensitivity of CCA cells. To this end, we employed the KKU-213 cell line that was shown to be very aggressive in a previous study [30]. As a representative of chemotherapeutics, we chose 5-FU because it is the one mostly used for the adjuvant therapy in our cohort of patients. We monitored the cell growth of KKU-213 cells incubated for up to 96 h in a medium from control or si-IL-6-transfected CAFs and exposed to 5-FU. SRB staining, which reflects the protein mass in the culture, indicated that cell growth was stimulated by the CAF-conditioned (scramble) medium while it was inhibited by the conditioned medium derived from si-IL6-transfected CAFs (Figure 7A). More importantly, the growth was greatly inhibited by 5-FU, and even more when the treatment was performed in the cells incubated with the CAF-conditioned medium lacking IL-6 (Figure 7A). The clonogenic assay confirmed that 5-FU could inhibit the proliferation of KKU-213 cells more effectively when incubated in the medium of CAFs deprived of IL-6 (Figure 7B,D). Cytofluorometer analysis further proved that this effect was not merely due to the blockade of cell proliferation and instead was due to induction of cell death, very likely apoptosis based on the hypodiploid subG1 peak (Figure 7C,E). When treated with 5-FU, the percentage of the subG1 population (referable to apoptotic cells) in the control medium was of approx. 33%, while in the CAF-derived medium it was approx. 23% (i.e., one-third less) and in the IL-6-deficient CAF-derived medium it was approx. 57% (i.e., it nearly doubled). Finally, we asked whether the CAF-derived effects on chemoresponsivity to 5-FU were linked to autophagy in CCA cells. We further investigated the link between the CAF secretions (from scramble si-RNA or si-RNA IL-6-transfected cells) and the autophagy-dependent response to 5-FU. To this end, we first looked at the expression of the autophagy proteins LC3 and p62, of the pro-apoptotic protein BAX, and of the anti-apoptotic protein BCL-2. Autophagy was assessed by looking at the ratio of conversion of LC3-I into LC3-II (LC3-II/LC3-I is a rough measure of autophagosome formation), the ratio of LC3-II/β-actin (a rough measure of the autophagosomes present in the cell), and the ratio of p62 (SQSTM1)/β-actin (a rough measure of the autophagy substrate being degraded). In drug-untreated cultures, compared with the protein expression in cells cultivated in standard medium, the IL-6-rich conditioned CAF medium limited the activation of autophagy (reduced the conversion of LC3-I into LC3-II; accumulation of p62) and reduced the expression of BAX, whereas the IL-6-deficient CAF medium (upon si-IL-6 transfection) greatly stimulated the autophagy flux, reduced the expression of BCL-2, and restored the expression of BAX (Figure 8A,B). On treatment with 5-FU, autophagy was stimulated along with increased expression of BAX in the cells cultivated in standard medium, consistent with the induction of a toxic stress-response and the onset of apoptosis (Figure 8A,B). The 5-FU stimulated not only autophagosome formation (LC3-I is converted into LC3-II) but also the autophagy flux, as indicated by decreased LC3-II and p62 levels (indicative of effective degradation). Remarkably, this response to 5-FU was largely impaired in the cells incubated with the IL-6-rich CAF medium (from scramble si-RNA-transfected cells), while it was enhanced when the cells were treated in the CAF medium lacking IL-6 (Figure 8A,B). To better assess the functional link between autophagy and apoptosis in response to 5-FU, we performed a double immunostaining for LC3 (to mark the cells with ongoing autophagy) and for BAX (to mark the cells undergoing apoptosis). The images in Figure 8C show that 5-FU can induce BAX-mediated apoptosis only in the cells cultivated in standard medium or in IL-6-deficient CAF medium, while it is not effective in cells cultivated in IL-6-rich CAF medium. The double staining also demonstrates that apoptosis (BAX-positive) ensued in the same cells in which autophagy (LC3-positive) was induced (Figure 8C). Further, we added the staining with Annexin-V-APC (to label apoptotic cells) and p62 in addition to LC3 (as markers of autophagy). Data are shown in panels D and E (representative fields are shown; ImageJ quantification is presented in Supplementary Figure S7). The data confirm that apoptosis associated with autophagy occurs in 5-FU-treated cells, and this event is more pronounced when the cells are incubated in IL6-deprived CAF CM. Particularly, p62 staining greatly decreases in the cells incubated with si-IL6 CAF medium, which proves that IL-6 impairs the autophagy flux. Finally, to definitively prove the involvement of autophagy and apoptosis in 5-FU toxicity and their inhibition by CAF-derived IL-6, we included the treatments with Spautin-1, a drug that induces the degradation of BECLIN-1 (thus inhibiting BECN1-dependent autophagy), and with z-VAD (OMe)-fmk, a pancaspase inhibitor that inhibits apoptosis. The expression of LC3 and of BAX was determined by immunofluorescence. The representative images in Figure 8F,G (ImageJ quantification is presented in Supplementary Figure S7) demonstrate that both treatments prevented cell death by 5-FU along with inhibition of autophagy (reduced LC3 staining) or apoptosis (reduced BAX staining) when compared with the untreated cells in panel A.

### 2.8. Impaired Autophagy by Stromal Inflammation in an Experimental Animal Model of Cholangiocarcinoma

**Figure 9:**
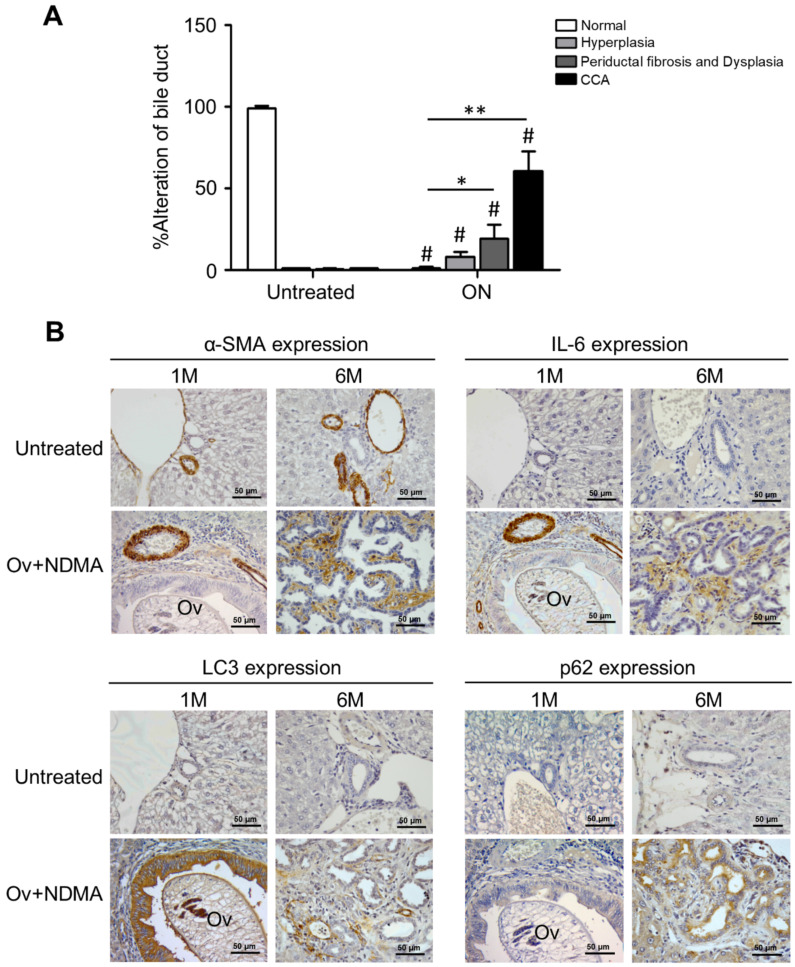
Correlation between stromal fibrosis and IL-6 with autophagy in cancer cells in an experimental animal model of cholangiocarcinoma. *Opistorchis-viverrini*-associated cholangiocarcinogenesis was induced in hamsters, and liver bile duct sections from animals at 1 month and 6 months post-treatment were prepared for histology and IHC analyses. (**A**) Histologic alterations were estimated by the pathologist as hyperplasia, fibrosis/dysplasia, and frank cholangiocarcinoma. Bile duct alteration scores of the ON and untreated groups. Significantly different at * *p* < 0.05 and ** *p* < 0.01. # Significant compared with the untreated group. (**B**) IHC of α-SMA (a marker of CAFs) and IL-6 in the stroma and of LC3 and p62 (markers of autophagy) in CCA cells. Apparent is the increased staining of α-SMA, IL-6, and p62 along with decreased staining of LC3 in the sections from ON-treated hamsters at Month 6. Representative images are shown (Magnification 40×).

To further support our contention of an inhibitory activity of inflammatory stroma on autophagy regulation in CCA cells, we employed a model well established to reproduce the natural cholangiocarcinogenesis in humans by *Opistorchis viverrini* (Ov) [35]. In this experimental model (ON), hamsters are infected with *O. viverrini* and treated with *N*-dinitrosomethylamine (NDMA) to develop a CCA as demonstrated by the alterations in bile duct epithelial tissue that shows progressive hyperplasia followed by periductal fibrosis and dysplasia and ending in frank cholangiocarcinoma (Figure 9, panel A). We detected by IHC the markers for autophagy (LC3 and p62) and for activated stromal fibroblasts (CAFs; with α-SMA) and for IL-6 in tumor specimens at Month 1 and 6 after cholangiocarcinogenesis, i.e., at early and late stages of stromal fibrosis. At the late stage, bile duct alterations are much evident in the ON-treated hamsters (panel A). The IHC staining in Figure 9 (panel B) shows that compared with the pattern at M1, at M6 the staining for α-SMA and for IL-6 is much more intense and in parallel the staining of p62 (a marker of autophagy degradation) increases along with decreased staining of LC3 (a marker of the autophagosome), which, taken together, indicate the downregulation of the autophagy pathway. The expression of LC3, p62, and IL-6 was not detectable in the bile ducts in the untreated group (Figure 9B).

## 3. Discussion

CCA is a prototype of inflammatory cancer with a highly desmoplastic stroma particularly rich in activated CAFs with a myofibroblast-like phenotype [36,37]. Fibroblasts isolated from CCA tissues have been shown to release a range of soluble factors with pro-tumorigenic activity [38]. Proinflammatory cytokines, particularly IL-6, secreted by both cancer cells and CAFs determine a malignancy-driven tumor microenvironment that promotes CCA progression [39]. Consistently, CCA cells exhibit very aggressive behavior, both in vitro and in vivo, when exposed to IL-6 [40,41,42,43,44,45]. IL-6 may contribute to CCA malignancy through a variety of pathways, including the inhibition of autophagy in CCA cells [30,41]. Autophagy (precisely, macroautophagy) is a quality-control process that directs redundant, aged, and non-functional cell components to lysosomes for their complete degradation [46]. Autophagy runs regularly at the basal level to maintain cell homeostasis and can be upregulated in response to endogenous or exogenous stresses [47]. Insufficient autophagy has been shown to favor carcinogenesis and, on the other hand, its upregulation may confer a survival advantage to cancer cells exposed to genotoxic, proteotoxic, and metabolic stresses [48]. Thus, to exploit autophagy as a therapeutic target in cancer management, it is fundamental to determine how it is dysregulated within the tumor [49].

It is now accepted that the actual level and functional outcome of autophagy in cancer cells depend on several factors [50], precisely: (1) the genetic background (i.e., the mutation in relevant oncogenes and tumor suppressor genes as well as in genes controlling the regulatory pathways) of the cancer cell; (2) the presence of growth factors, and the level of inflammation and of secreted inflammatory factors in the TME; (3) the structural and metabolic status of the TME (vascularization, availability of nutrients and of oxygen); and (4) the presence of epigenetic modifiers in the TME. Further, autophagy is a dynamic process that adjusts its regulation in the cells depending on the microenvironment. Therefore, the expression of autophagy markers reflects the situation in the tumor mass context at the time of the analysis.

The literature on the involvement of autophagy in CCA development and progression reports some contradictory data about its pro-tumorigenic or anti-tumorigenic role [49]. For instance, in vitro experiments with CCA cells showed that disruption of BECLIN-1-dependent autophagy could either sensitize to or abrogate cytotoxicity by chemotherapeutic drugs [26,30]. On the other hand, data from CCA xenograft experiments in nude mice might not truly resemble the situation in humans because of the lack of an efficacious immune–inflammatory response. Indeed, the uncertainty about the role of autophagy in cholangiocarcinogenesis and the therapeutic response and clinical outcome likely reflects our lack of knowledge about the functional relationship between the level of inflammation in the stroma and the actual level of autophagy in CCA cells. The present study attempts to fill in this gap of knowledge. Briefly, we found that: (1) high IL-6 in CAF-containing fibrotic areas was significantly associated with a shorter overall survival; (2) the combined pattern of high LC3 and low p62 in CCA cells showed a significant correlation with the best overall survival; (3) the pattern of L/H/L, representing a low inflammatory stroma (low IL-6 staining) and an efficient autophagy flux in cancer cells (high LC3 and low p62), was significantly associated with the best prognostic clinical outcome; and (4) the tumors with low stromal IL-6 and high cancer cell LC3 expression were more responsive to chemotherapy than tumors with any other combination. To generalize the conclusion of our study, we interrogated the TCGA database for CCA. However, the limited number of worldwide cases and the lack of complete information on the expression of autophagy markers in the database did not allow us to draw statistically significant conclusions regarding their prognostic value, though the available data consistently suggest that increased autophagy flux (with high LC3 and low p62) associates with a better prognosis. In apparent contradiction, the TCGA data also indicate that CCA with high expression of BECN1 (one of the most important autophagy genes) had a worse prognosis. However, a more in-depth analysis revealed that many of these patients were not subjected to chemotherapy. In addition, many CCAs with high *BECN1* mRNA indeed expressed a low level of *LC3* mRNA, indicating that autophagy in those samples was not active. In fact, the functional link between BECLIN 1 autophagy and clinical outcome should be based on a true assessment of autophagy at the protein level, with particular attention paid to the expression of BECLIN 1, LC3, and p62 along with that of BCL-2 (an inhibitory interactor of BECLIN 1 that confers apoptosis resistance) in the same cells [51]. Supporting this interpretation, it was found that CCAs with low expression of BECLIN 1 were significantly associated with lymph node metastasis and worse OS, while cases with no lymph node invasion showed the highest expression of BECLIN 1 [24,32]. Higher levels of LC3, BECLIN 1, and p62/SQSTM1 proteins were found in specimens of CCAs at all steps of progression, from pre-invasive to invasive carcinomas, compared with non-neoplastic large bile duct and peribiliary gland tissues [25]. The concomitant high expression of both LC3 and p62 could be interpreted as a defective autophagy flux since the very early steps of cholangiocarcinogenesis, which would be consistent with a tumor-suppressive role of autophagy in cholangiocarcinogenesis. We have tested this hypothesis in an experimental animal model where hamsters infected with Ov and treated with NMDA develop a cholangiocarcinoma in a fashion resembling the Ov cholangiocarcinogenesis in humans (Figure 9) [35]. Consistent with our previous data [52], we found that LC3 is induced during cholangiocarcinogenesis. However, at a late stage when CCA is dominated by highly fibrotic areas, enriched in IL-6 producing CAFs, the expression of LC3 decreases while that of p62 increases, indicating an impairment of the autophagy process in cancer cells. Thus, again, it appears fundamental to clearly assess the efficiency of the autophagy flux before drawing conclusions about the functional role of this process in cancer progression and chemotherapy response. To better understand this aspect, we attempted to reproduce (at best) in vitro the effect of IL-6 on the chemotherapeutic response in human CCA cells and to assess the role of autophagy in this response. Primary CAFs were isolated from human CCA and genetically impaired to produce IL-6 through specific siRNA transfection. CCA cells were then exposed to 5-FU (a prototype chemotherapeutic for CCA treatment) diluted in the conditioned medium of control (scramble-siRNA-transfected) or of IL-6-depleted (IL-6-siRNA-transfected) CAFs, and cell growth, colony formation, and apoptosis were assessed. The IL-6-rich medium from CAFs stimulated CCA growth and prevented 5-FU cytotoxicity along with inhibition of autophagy. Interestingly, 5-FU was more effective when administered in CCA cultures devoid of IL-6, and BAX-mediated apoptosis was shown to occur in CCA cells accumulating LC3-positive vacuoles. This outcome can be explained by the two-hit model [53], which predicts that in cells with active autophagy (the first stressing hit) any additional stress (in this case, the chemotherapeutic) further stimulates the autophagy precipitating autophagy-associated apoptosis. This sequence of events in 5-FU-treated CCA and their inhibition by IL-6 was further confirmed by inhibiting BECLIN-1-dependent autophagy with Spautin-1 and by inhibiting apoptosis with the pancaspase inhibitor z-VAD (OMe)-fmk.

One limitation of the present study is the relatively small cohort of CCA patients analyzed. This might be the reason for the lack of statistical significance between clinical data and clinical outcome.

Nonetheless, we found a statistically significant correlation between the production of IL-6 by CAFs and the inhibition of autophagy in CCA cells, which reflected in a poor prognosis. These findings were corroborated with in silico data from the TCGA and with experimental data from in vitro and animal CCA models. Taken together, these data are the first to show a functional and mechanistic correlation between stromal inflammation and response to the chemotherapy in cholangiocarcinomas. As such, this study can be considered a ‘proof-of-concept’ that shall be validated in a larger cohort and applied to other cancers as well.

Our study has several clinical implications. First, it confirms the need to determine the level of stromal inflammation (namely, the level of CAFs and of IL-6) to better design the therapy and predict the prognosis. Second, in view of a personalized treatment, assessing autophagy thoroughly with several markers is mandatory for a better stratification of the patients that could benefit from drugs specifically targeting autophagy. Third, it supports the inclusion of autophagy-enhancing drugs to improve the clinical response. In this regard, some natural products such as pristimerin [54], dihydroartemisinin [30], pterostilbene [55], and resveratrol [41] seem very promising. We propose to use such autophagy-enhancing drugs along with navitoclax, which has been shown to kill CAFs in CCA [23], or with resveratrol, which has been shown to switch off the synthesis of IL-6 by CCA-derived CAFs [41].

## 4. Materials and Methods

### 4.1. Patients, Samples, and Ethical Issues

The CCA tissue microarrays (CCA-TMAs) of paraffin-embedded cases originated from primary tumors of 70 patients who were admitted to surgical wards of Srinagarind Hospital, Khon Kaen University, Khon Kaen, Thailand, collected between 2014 and 2016.

### 4.2. Cell Lines and Primary Cancer-Associated Fibroblasts

The human CCA cell line KKU-213 was obtained from the Liver Fluke and Cholangiocarcinoma Research Center, Khon Kaen University (Thailand). The KKU-213 cell line was maintained under standard culture conditions in Ham’s F-12 medium supplemented with 10% heat-inactivated FBS, 1% Glutamine (Sigma–Aldrich), and 1% Penicillin/Streptomycin (Sigma–Aldrich) (referred to as CoM, control medium) in an atmosphere of 5% CO_2_ at 37 °C. The primary culture of CCA-associated fibroblasts (CAFs) was isolated using a standard procedure from the CCA of a patient who underwent surgery at Srinagarind Hospital, Khon Kaen University (Thailand). CAFs were cultivated in Ham’s F-12 media containing 10% FBS for 10–15 days to allow for the formation of colonies (designated as passage 0). CAF cells were sub-cultured when 80% confluent, banked, and used for experimental studies at passages 5–13.

### 4.3. Antibodies and Reagents

The primary antibodies used for immunohistochemistry (IHC), immunofluorescence (IF), and Western blotting (WB) were purchased from Abcam (Cambridge, MA, USA), anti-LC3 (ab51520, IHC 1:500; WB 1:1000; IF 1:100), anti-p62 (ab91526, IHC 1:500; WB 1:500; IF 1:100), anti-IL-6 (ab6672, IHC 1:1000; WB 1:1000; IF 1:100), anti-BAX (ab3191,WB 1:2000; IF 1:100), and anti-BCL-2 (ab196495, WB 1:1000; IF 1:100). Peroxidase-conjugated Envision^TM^ IHC secondary antibody was purchased from DAKO (K4001), Denmark. Horse radish peroxidase (HRP)-conjugated goat anti-mouse (1:2000) and goat anti-rabbit (1:2000), both from BioRad (Hercules, CA), were used as secondary antibodies for WB. Alexa Fluor^TM^ 555 goat anti-mouse IgG (red fluorescence, Abcam) from Life technologies or Alexa Fluor^TM^ 488 goat anti-rabbit IgG (green fluorescence, Abcam) or Alexa Fluor^TM^ 594 goat anti-mouse IgG (red fluorescence, Abcam) from Invitrogen, both from Thermo Fisher Scientific Co., Ltd, Waltham, MA, USA, was used for IF. Annexin V-APC (31490016, ImmunoTools, Germany) was used to identify apoptotic cells. Hoechst, trihydrochloride trihydrate, from Invitrogen was added to stain the nucleus. Spautin-1 (SP-1, 1262888-28-7) was purchased from Sigma-Aldrich and used at 5 μM [56]. The pancaspase inhibitor z-VAD (OMe)-fmk (z-VAD-fmk, Alexis Laboratories, San Diego, CA, USA) was dissolved in DMSO to prepare the stock solution and thereafter diluted in the culture medium at a final concentration of 10 μM. Control experiments demonstrated that DMSO (with a final concentration of 0.01%) had no effect on cell growth and autophagy.

### 4.4. Cytotoxicity Assay

Cells were plated in triplicate in 96-well plates at 1 × 10^3^ cells confluence per well. Twenty-four hours later, the cells were treated with CAF CM with or without 5-FU or their combination for 48 h at the concentrations indicated. Cell viability was measured using a SRB assay. Cell viability numbers were determined by calculating the average OD from three wells and the experiment was repeated in triplicate.

### 4.5. Cell Viability

Cell viability was evaluated using a colorimetric assay. Cell viability was measured based on a colony formation assay stained with 0.5% crystal violet. Briefly, KKU-213 cells (200 cells/well) were cultured in six-well plates and incubated with 5-FU for 10 days. The medium and substances for treatment were renovated every 3 days (day 3, day 6, and day 9). At the end of the treatment, the cells were washed with 1× PBS, fixed with 10% TCA (trichloroacetic acid), stained with a 0.5% crystal violet solution, and washed with tap water until excess dye was removed. The colony number was counted by photometric measurements using the CellCounter software (Nghia, Ho) version 0.2.1. Three independent experiments were performed for each assay condition. Flow cytometry was performed as previously described using propidium iodide (PI, 50 µg/mL final concentration) (Alexis Laboratories, San Diego, CA, USA) and analyzed in a FacScan flow cytometer (Becton Dickinson, Franklin Lakes, NJ, USA).

### 4.6. Immunohistochemical Analysis

LC3, p62, and IL-6 were detected on the CCA-TMAs of 70 paraffin-embedded sections using standard immunohistochemistry protocols. In brief, tissue sections were deparaffinized in xylene and rehydrated in a series of concentrations of ethanol. Thereafter, antigen retrieval was performed by microwaving sections in 10 mM sodium citrate (pH 6) + 0.1% Triton X-100, 10 min for LC3 and p62 protein, and 10 mM sodium citrate (pH 6), 10 min for IL-6 protein antigen retrieval. Then, the sections were endogenous peroxidase activity blocked with 0.3% H_2_O_2_ for 30 min. Nonspecific binding was blocked by 10% skim milk in 1× phosphate-buffered saline (1× PBS) for 30 min. Sections were incubated with the primary antibody at 4 °C in a moisture chamber overnight. After that, sections were incubated with peroxidase-conjugated Envision secondary antibody (DAKO, Glostrup, Denmark). The color was developed with 0.1% diaminobenzidine tetrahydrochloride solution for 5 min and the sections were counterstained with Mayer’s hematoxylin. The CCA-TMA sections were observed under a light microscope (Eclipse, Ni-U, Nikon Instruments Inc., Melville, NY, USA) by using the high magnification power ×200. The staining frequency of proteins was semiquantitatively scored based on the percentages of positive cells, as follows: 0% = negative; 1–25% = +1; 26–50% = +2; and >50% = +3. The intensity of protein staining was scored as weak = 1, moderate = 2, and strong = 3 [57].

### 4.7. Western Blot Analysis

The KKU-213 cell line was lysed with radioimmuno-precipitation assay (RIPA) buffer containing protease inhibitor cocktails, 0.5 M NaF, 0.2 M NaVO_4_, 1 M Tris-HCl pH 7.5, 0.5 M EDTA, 2.5 M NaCl, 10% (*v*/*v*) NP-40, 10% (*w*/*v*) SDS, Triton X-100, and deionized water. A protein assay was performed using bicinchoninic acid (BCA; Thermo ScientificTM, Rockford, IL, USA). A total of 20 µg of liver homogenate was fractionated by SDS-PAGE and transferred to a polyvinylidene fluoride membrane (Whatman, Dassel, Germany). The membranes were incubated overnight at 4 °C with the primary antibody, followed by incubation with the appropriate secondary antibody at room temperature for 1 h in Enhanced Chemiluminescence Plus solution (GE Healthcare, Buckinghamshire, UK). The band intensity was quantified with Image Quant Imager and ImageQuant analysis software (GE Healthcare, Uppsala, Sweden). The intensity of the bands was estimated by ImageJ software (NIH, Bethesda, MD, USA). The membranes were also stained for β-actin as an internal control of protein loading.

### 4.8. Immunofluorescence Staining

CCA cells on coverslips were incubated for double immunofluorescence staining with the indicated primary antibody followed by the appropriate secondary fluorescent antibody. Hoechst was added to stain the nucleus. Antibodies were diluted in 0.1% Triton X-100 in 1× PBS + 10% FBS. Fluorescence was captured with a Carl ZEISS 710 confocal fluorescence microscope (Carl Zeiss Microscopy GmbH, Jena, Germany) equipped with ZEN Software.

### 4.9. Transient Knockdown of Small Interfering RNA for IL-6 into CAFs

CAFs (1 × 10^5^ cells) isolated from CCA tissues were seeded in a six-well plate for 24 h prior to transfection. Small interfering RNA (si-RNA) transfection was performed using Lipofectamine RNAiMAX (Invitrogen). Stealth RNAi si-RNA for IL-6 (Invitrogen), or a stealth RNAi si-RNA negative control (Invitrogen), was transfected at a final concentration of 5 nM. After 24 h of transfection, media were changed to 10% FBS HAM F’12 and cultured for 24 h. CAF CM was collected by growing CAFs for 2 days until CAFs reached about 80% confluence. Then, CAF CM was centrifuged at 1000× *g* for 10 min to remove cell debris, sterile filtered, and stored at −80 °C until used.

### 4.10. The Cancer Genome Atlas (TCGA)

We have collected the clinical data on cholangiocarcinomas from cBioportal publicly available online platforms (www.cbioportal.org; accession date: 27 March 2020). The Cancer Genomic Atlas Project (TCGA, Firehose Legency) reports a total number of 34 cholangiocarcinoma patients/samples (including intrahepatic, extrahepatic, and perihilar cancer subtypes). We extracted the information related to messenger RNA (mRNA) expression (at https://genome-cancer.ucsc.edu/proj/site/hgHeatmap; accession date: 27 March 2020), and we analyzed the cross-comparison between pairs of genes based on mRNA expression groups for BECN1, MAP1-LC3B, p62/SQSTM1, and IL6. All statistical analyses were performed using Excel and SAS software (9.4. version, SAS Institute Inc., Cary, NC, USA) following SAS/STATs and are represented in box plot format. The *p*-values <0.05 were considered significant.

### 4.11. Animal Model, Treatments, and Histological Analyses

CCA carcinogenesis was induced in Syrian golden hamsters by infection with Ov metacercariae combined with NDMA treatment as previously described [35]. Hamsters were divided into two groups: (A) untreated control (n = 10) and (B) *O. viverrini* plus NDMA (ON group). The latter were infected with 50 *O. viverrini* metacercariae by intragastric intubations, and NDMA was administered in drinking water (12.5 ppm) provided ad libitum for 2 months. Hamsters were euthanized and the tumors removed at 1 and 6 months, and liver tissue was collected for histology examination by the pathologists. For tissue sections, excised liver specimens were cut and fixed in 10% buffered formalin for 24 h. The fixed tissues were embedded in paraffin and then serially dissected into 4 µm thick slides. Biliary epithelial alterations were graded as hyperplasia, dysplasia ducts, periductal fibrosis, and CCA as described previously [58]. IHC for autophagy markers, stromal CAFs, and IL-6 was performed as described above.

### 4.12. Statistical Analysis

The relationship between IL-6 and autophagy protein expression and clinicopathological factors was analyzed using an χ2 test, Fisher’s exact test, or a Kruskal–Wallis test. OS was defined as the period from the date of surgery to the date of death or the last day of follow-up. Survival curves were analyzed with the Kaplan–Meier (KM) method and compared using the log-rank test. Numbers at risk are reported in relevant KM curves. Multivariate analysis of putative prognostic factors was evaluated in a Cox proportional hazards model. The correlation between two continuous variables was calculated using Pearson’s correlation coefficient (r). Data are presented as mean ± SD. All analyses were two-sided, and *p*-values less than 0.05 were considered statistically significant. Statistical analyses were performed using SPSS 20.0 statistics software (SPSS Inc., Chicago, IL, USA).

## 5. Conclusions

This is the first demonstration that the expression of IL-6 in the stroma and of autophagy markers in cancer cells may have prognostic value and help to stratify responder and not responder patients bearing a CCA. The results of this study collectively suggest that a therapeutic strategy of autophagy-enhancing drugs along with chemotherapeutics to fight the stromal inflammation could improve the clinical outcome of CCA patients.

## Figures and Tables

**Table 1:** Clinicopathological variables of IL-6 in CAFs, LC3, and p62 in cancer cells of 70 CCA samples in the study cohort.

Factor	*n*	LC3		*p*	P62		*p*	IL-6 (in CAFs)		*p*
		Low	High		Low	High		Low	High	
Age (years)										
≤55	35	16	19	0.316	27	8	0.500	20	15	0.500
>55	35	19	16		26	9		19	16	
Sex										
Female	27	13	14	0.500	22	5	0.275	16	11	0.412
Male	43	22	21		31	12		23	20	
Histological types										
Non-pap	33	18	15	0.316	26	7	0.388	17	16	0.335
Papillary	37	17	20		27	10		22	15	
Tumor staging										
I/II	39	17	22	0.168	29	10	0.496	22	17	0.544
III/IV	31	18	13		24	9		17	14	
Tumor size (T stage)										
T1/T2	40	18	22	0.235	32	8	0.246	22	18	0.542
T3/T4	30	17	13		8	9		17	13	
Metastasis to lymph nodes (N stage)										
N0	44	23	21	0.342	33	11	0.099	25	19	0.412
N1/N2	26	12	14		20	6		14	12	
Metastasis to organs (M stage)										
M0	65	34	31	0.178	51	14	0.088	37	28	0.391
M1	5	1	4		2	3		2	3	
Resection status										
R0	49	24	25	0.500	37	12	0.604	26	23	0.339
R1	21	11	10		16	5		13	8	
Drug regimen after surgical resection										
No	46	24	22	0.401	38	8	0.604	30	16	0.025 *
Yes	24	11	13		15	9		9	15	
Recurrence after surgical resection										
No	51	23	28	0.141	36	15	0.088	29	22	0.480
Yes	19	12	7		17	2		10	9	

Correlation between IHC score and clinicopathological characteristics by Fisher’s exact probability test. * *p*-values less than 0.05 were considered statistically significant.

**Table 2:** Multivariate Cox regression model for disease-free survival including IL-6 in CAFs and LC3 and p62 in cancer cells.

Variable (No. of Patients)	No. of Patients	Hazard Ratio (HR)	95% Confidence Interval (CI)	*p*-Value
LC3 (in cancer) IHC score				
Low	35	1		
High	35	0.401	0.236–0.681	0.001 **
p62 (in cancer) IHC score				
Low	53	1		
High	17	0.699	0.400–1.221	0.208
IL-6 (in CAFs) IHC score				
Low	39	1		
High	31	2.004	1.138–3.527	0.016 *
Age (years)				
≤55	35	1		
>55	35	0.505	0.308–0.828	0.252
Sex				
Female	27	1		
Male	43	0.844	0.507–1.407	0.516
Histological types				
Non-papillary	33	1		
Papillary	37	1.174	0.725–1.903	0.514
Tumor stages				
I–II	39	1		
III–IV	31	0.535	0.330–0.869	0.012 *
T stages				
T1/T2	40	1		
T3/T4	30	1.217	0.954–1.553	0.114
N stages				
N0	44	1		
N1/N2	26	0.792	0.494–1.269	0.332
M stages				
M0	65	1		
M1	5	0.604	0.226–1.612	0.314
R stages				
R0	41	1		
R1	29	1.298	0.874–1.928	0.196
Drug regimen after surgical resection				
No	34	1		
Yes	36	1.192	0.551–2.581	0.655

Multivariate analysis by Cox proportional hazard regression. CI 95% indicates the 95% confidence interval. * *p*-values less than 0.05 and ** *p*-values less than 0.01 were considered statistically significant.

**Table 3:** Pearson correlation coefficients between IHC scores of t-drug receivable, IL-6 in CAF, and LC3 and p62 in cancer cell (combined model) components in human CCA tissues.

	Drug Regimen	Low IL-6/High LC3/Low p62
Drug regimen Pearson Correlation	1	0.898 **
Sig. (2-tailed)		0.000
*N*	70	70
Low IL-6/High LC3/Low p62 Pearson Correlation	0.898 **	1
Sig. (2-tailed)	0.000	
*N*	70	70

** Correlation is significant at the 0.01 level (2-tailed).

## Data Availability

The data presented in this study are available in this article and Supplementary Materials.

## Supplementary Materials

The following are available online at https://www.mdpi.com/article/10.3390/cancers13092134/s1, Table S1: Patients’ clinical characteristics from the TCGA Cholangiocarcinoma database; Table S2: The quantification of Western blot for Figure 8; Table S2: The log-rank *p*-value of interest; Figure S1: The flowchart of the cohort of patients included in the study with the level of expression of IL-6, LC3, and p62, and the chemotherapeutic treatment; Figure S2: TCGA database analysis of 34 CCA tissues and corresponding clinical data; Figure S3: The oncoprint showing the alterations in p62/SQSTM1 gene expression in 34 CCAs from the TCGA database; Figure S4: The oncoprint showing the alterations in IL-6 gene expression in 34 CCAs from the TCGA database; Figure S5: The whole Western blot for Figure 6; Figure S6: The whole Western blot for Figure 8; Figure S7: ImageJ quantification of the immunofluorescence staining shown in Figure 8.

## Abbreviations

CAFs	cancer-associated fibroblasts
CCA	cholangiocarcinoma
CoM	control medium
CI	confidence interval
HR	Hazard ratio
IL-6	interleukin-6
NDMA	*N*-nitrosodimethylamine
Ov	*Opisthorchis viverrini*
TCGA	the cancer genome altos
TMA	tumor microarray
