# Supplementary material for: Cancer-Associated Fibroblast-Derived IL-6 Determines Unfavorable Prognosis in Cholangiocarcinoma by Affecting Autophagy-Associated Chemoresponse
Source: Cancers (Basel). 2021 Apr 28;13(9):2134. doi: 10.3390/cancers13092134 (PMC8124468; doi:10.3390/cancers13092134)
Supplement: Supplementary file 1 [file cancers-13-02134-s001.zip › cancers-1169722-supplementary.pdf]

cancers

MDPI

Article

Supplementary Material: Cancer-Associated Fibroblast-Derived IL-6 Determines Unfavorable Prognosis in Cholangiocarcinoma by Affecting Autophagy-Associated Chemoresponse

Suyanee Thongchot [1,2,3], Chiara Vidoni [2], Alessandra Ferraresi [2], Watcharin Loilome [1,4], Narong Khuntikeo [4,5], Sakkarn Sangkhamanon [4,6], Attapol Titapun [4,5], Ciro Isidoro [2,*], and Nisana Namwat [1,4,*]

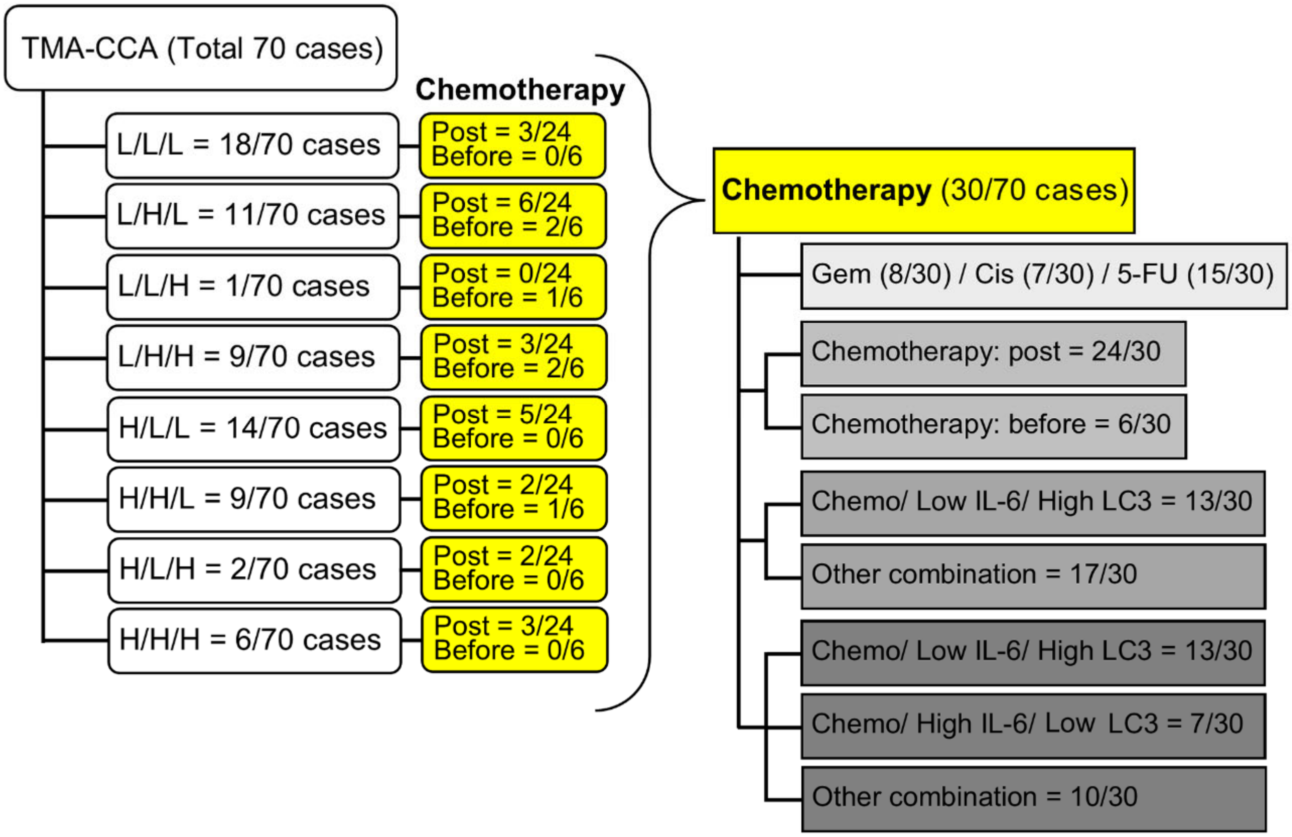

Figure S1. The flowchart of the cohort of patients included in the study with the level of expression of IL-6, LC3, and p62, and the chemotherapeutic treatment.

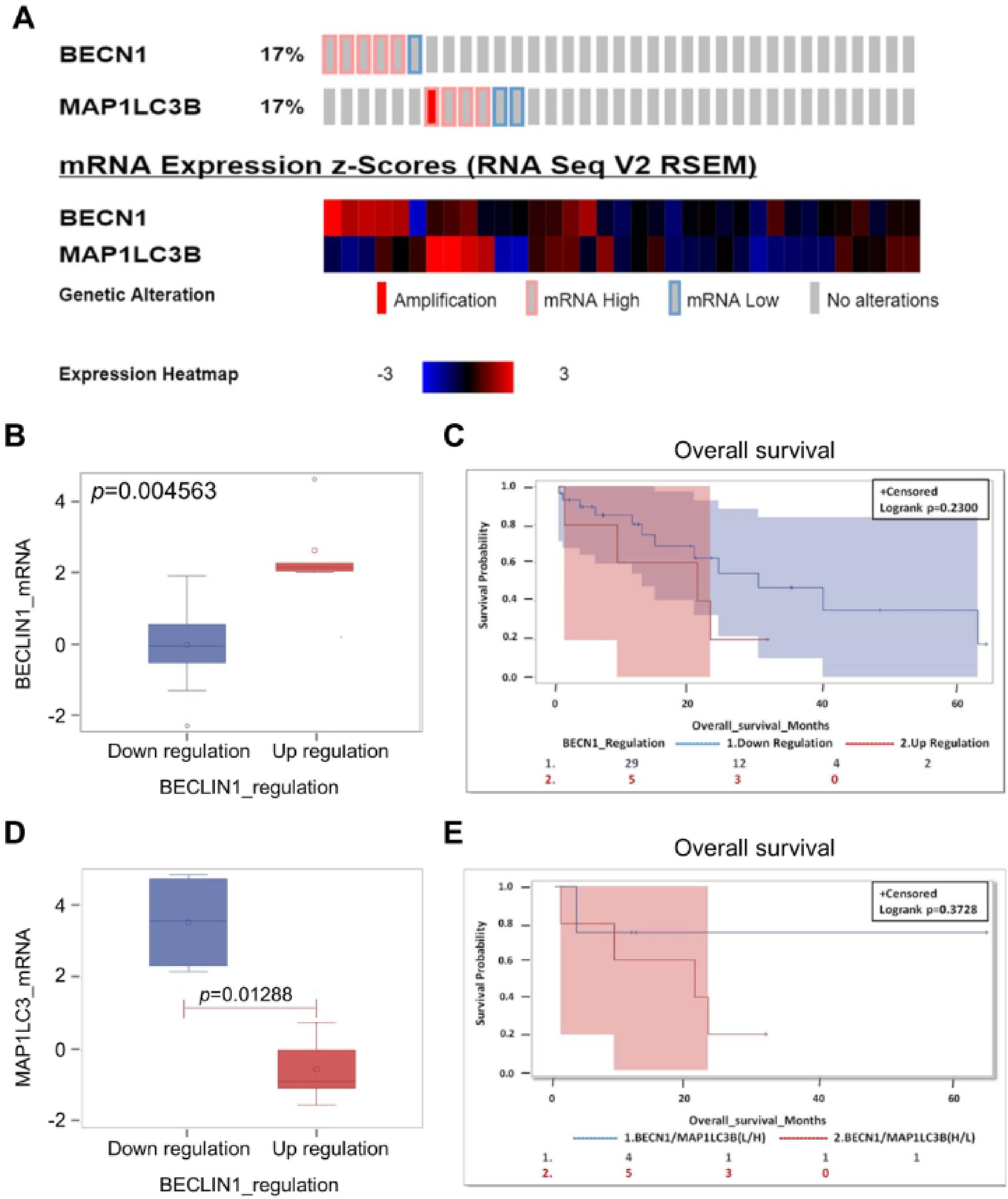

Figure S2. TCGA database analysis of 34 CCA tissues and corresponding clinical data

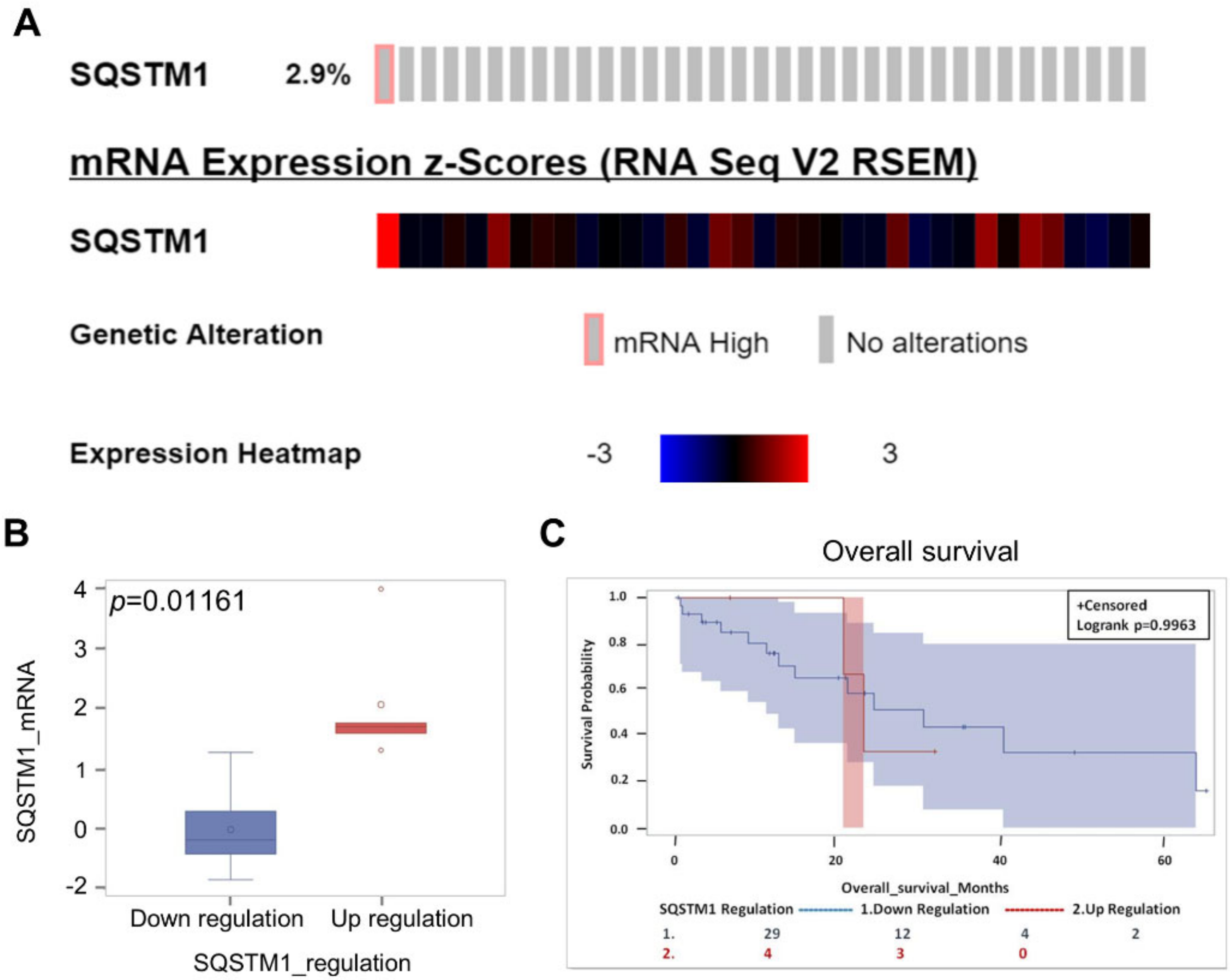

Figure S3. The oncoprint showing the alterations in p62/SQSTM1 gene expression in 34 CCAs from the TCGA database

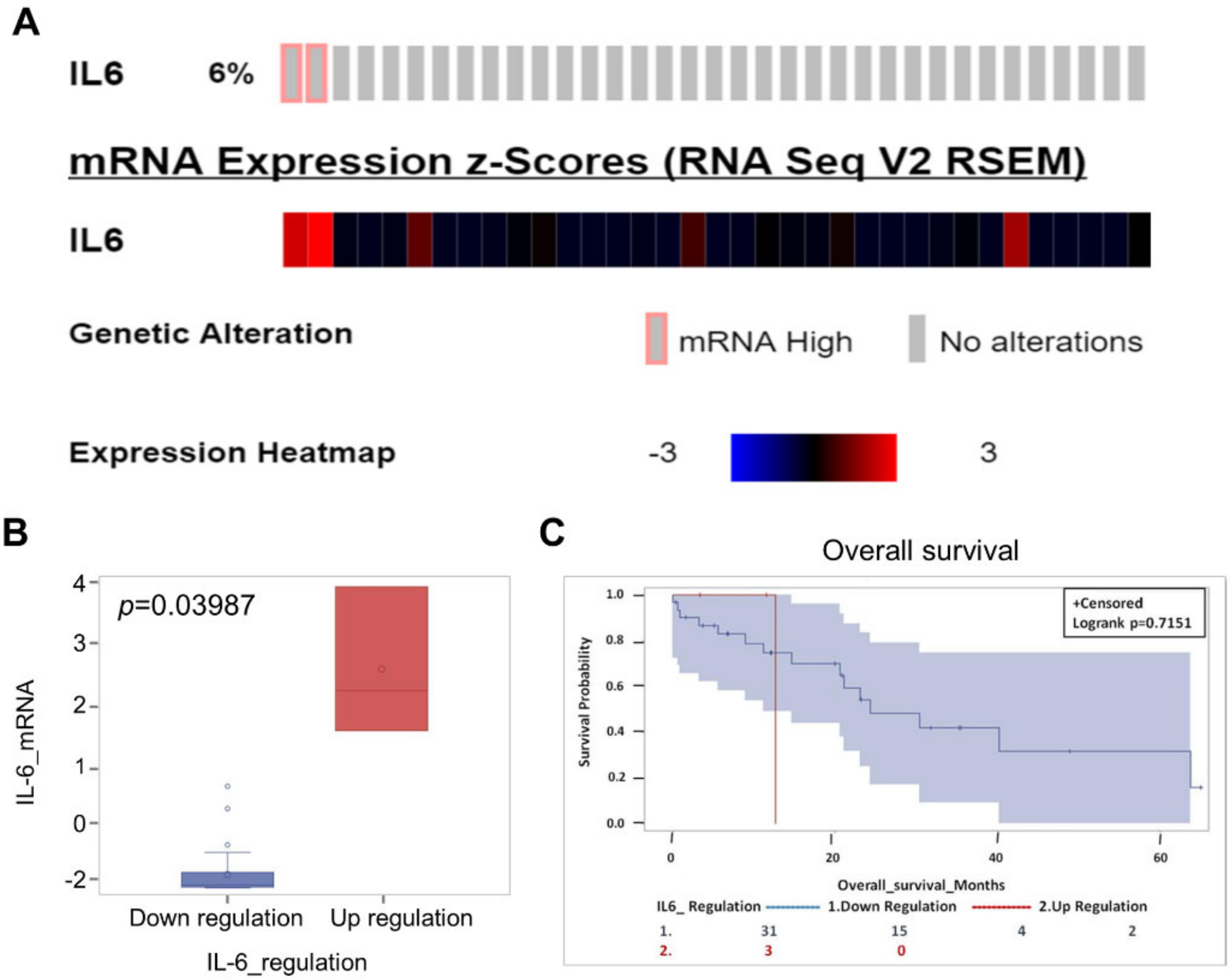

Figure S4. The oncoprint showing the alterations in IL-6 gene expression in 34 CCAs from the TCGA database

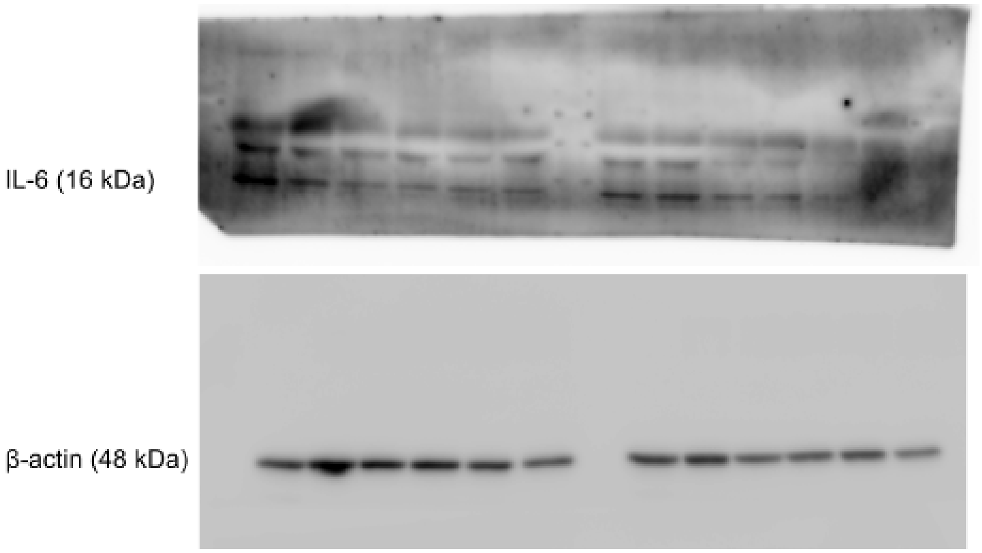

Figure S5. The whole Western blot for Figure 6

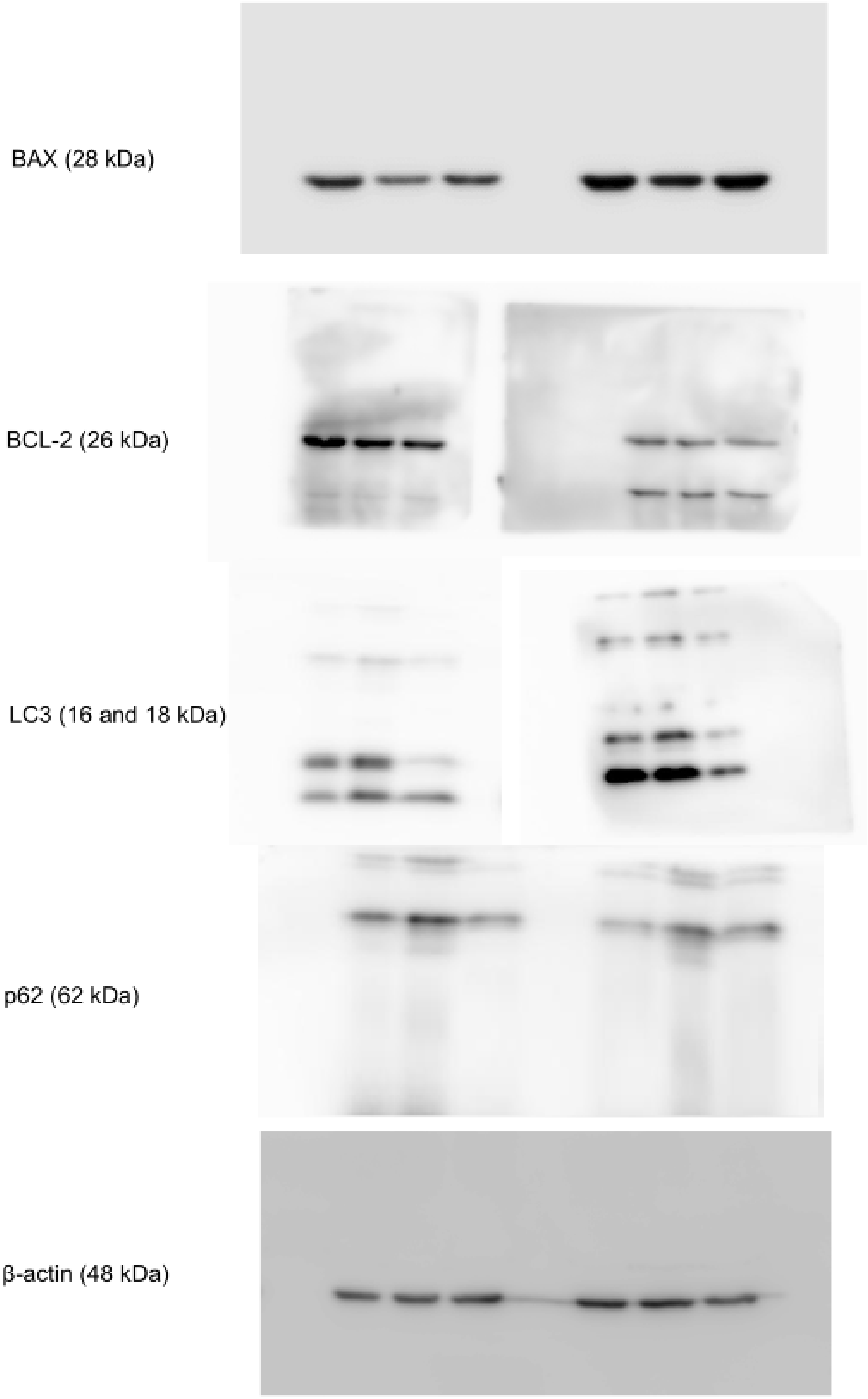

Figure S6. The whole Western blot for Figure 8

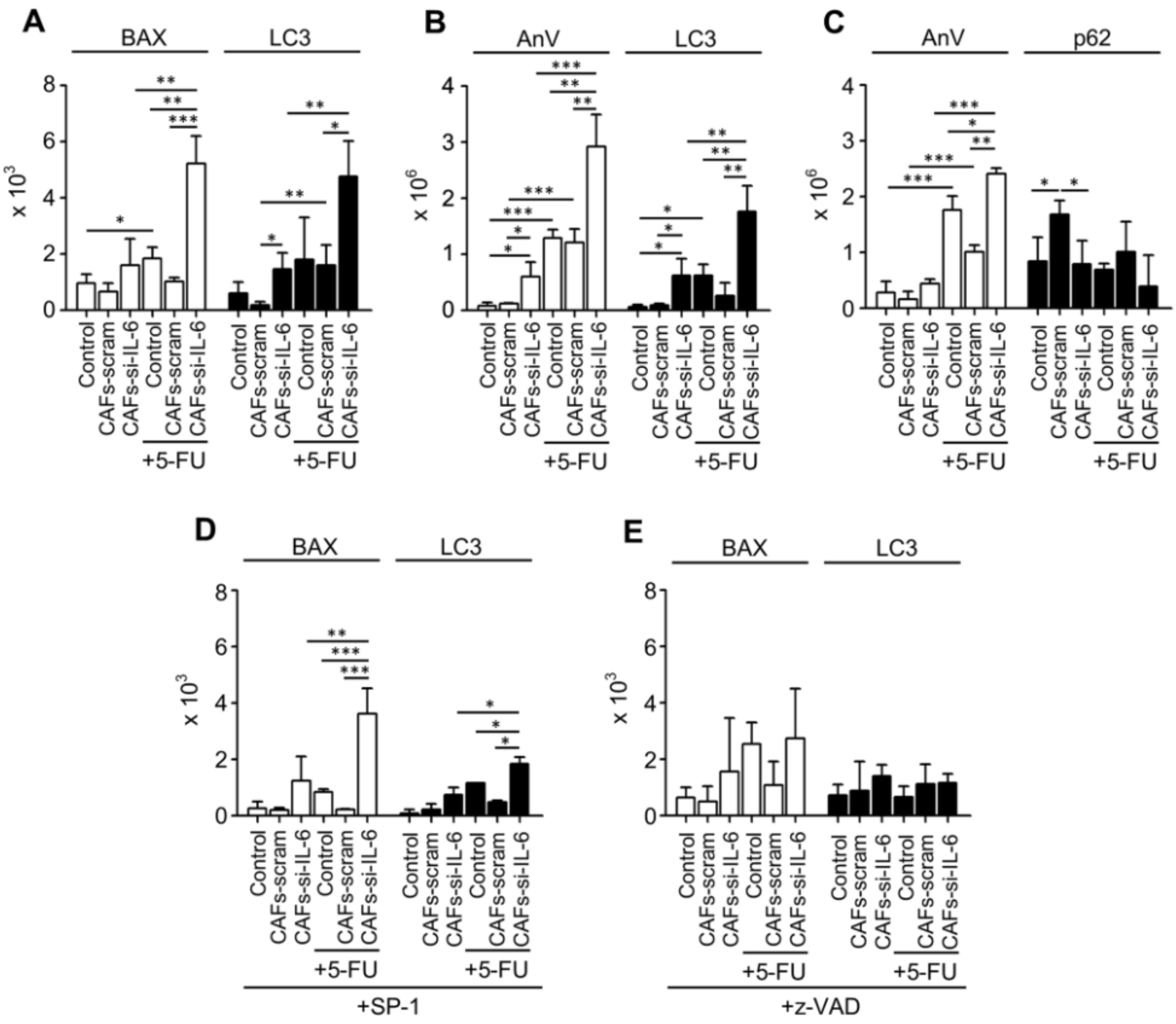

Figure S7. ImageJ quantification of the immunofluorescence staining shown in Figure 8.

Table S1. Patient's clinical characteristics of TCGA Cholangiocarcinoma database.

Characteristics	n = 35	MAP1LC3B		BECN1		SQSTM1		IL6	
		High(4)	Low(29)	High(5)	Low(29)	High(4)	Low(29)	High(3)	Low(31)
Age									
>55	27	2	23	3	23	3	22	2	24
<55	8	2	6	2	6	1	7	1	7
Sex									
Male	16	1	14	3	13	1	15	1	15
Female	19	3	15	2	16	3	14	2	16
Histology types									
Distal	2	-	2	-	2	-	2	-	1
Hilar/Perihilar	3	1	2	2	1	-	3	-	3
Intra-hepatic	30	3	25	3	26	4	24	3	27
Grade									
G1-G2	16	1	14	2	14	2	13	3	12
G3-G4	19	3	15	3	15	2	16	-	19
TNM Stage									
Stage I-II	28	4	22	3	24	4	22	2	26
Stage III-IV	7	-	7	2	5	-	7	1	5
Overall Survival									
Deceased	17	1	14	4	12	2	13	1	16
Living	18	3	15	1	17	2	16	2	15
Chemotherapy									
Yes	9	2	7	-	9	-	9	1	8
No	23	2	20	5	17	4	18	2	21
NA	3	-	2	-	3	-	2	-	2
Disease Free status									
Recurred/progressed	16	1	14	4	11	3	12	1	14
Tumor Free	15	3	12	-	15	1	14	2	13
NA	4	-	3	1	3	-	3	-	4

Table S2. The quantification of western blot in Figure 8A.

Condition				LC3-II/LC3-I ratio		LC3-II/LC3-I ratio
	media	LC3-I	7735.418		7989.452	
		LC3-II	4149.569	0.536438	4290.322	0.536998
	scram-CAFs	LC3-I	8792.1		7931.221	
		LC3-II	4291.334	0.48809	4134.56	0.521302
	si-IL-6-CAFs	LC3-I	1837.962		1906.541	
		LC3-II	4005.196	2.179151	3576.98	1.876162
5FU	media	LC3-I	2945.376		3152.598	
		LC3-II	7228.317	2.454124	6049.56	1.918913
5FU	scram-CAFs	LC3-I	3703.841		3235.184	
		LC3-II	6168.51	1.665436	6727.004	2.079327
5FU	si-IL-6-CAFs	LC3-I	1943.326		1903.167	
		LC3-II	6012.711	3.094031	3025.329	1.589629

Table S3. The significantly log-rank p-value of interest.

Figure	correlation	p-value
Figure 1B	IL-6 in cancer	0.886
	IL-6 in CAFs	0.024*
	IL-6 in cancer and CAFs	0.953
Figure 2B	LC3 in cancer	0.001***
Figure 2C	p62 in cancer	0.205
Figure 2D	Autophagy (LC3 and p62) in / cancer	0.001***
	-/- (blue line)	0.160
	-/+ (black line)	0.907
	+/- (green line)	0.010*
	+/+ (violet line)	0.544
Figure 3	IL-6 in CAFs and autophagy (LC3 p62) in cancer	0.007
	L/L/L (blue line)	0.047*
	L/H/L (green line)	0.002**
	L/L/H (light brown line)	0.224
	L/H/H (violet line)	0.159
	H/L/L (yellow line)	0.370
	H/H/L (red line)	0.176
	H/L/H (light blue line)	0.937
	H/H/H (gray line)	0.879
Figure 4A	Chemotherapy/IL-6/autophagy (LC3/p62)	0.001***
	No/others (blue line)	0.130
	Yes/others (green line)	0.054
	No/low/high/low (yellow line)	0.551
	Yes/low/high/low (violet line)	0.01**
Figure 4B	Chemotherapy/IL-6/LC3	0.001***
	Others (blue line)	0.370
	Chemo/low IL-6/High LC3 (green line)	0.001**
Figure 4C	Chemotherapy/IL-6/LC3	0.018*
	Others (blue line)	0.551
	Chemo/low IL-6/high LC3 (green line)	0.001**
	Chemo/high IL-6/low LC3 (yellow line)	0.130
